# Lifestyle and environmental risk factors for myopia in children: Evidence from a large-scale cross-sectional study in Shandong, China

**DOI:** 10.1371/journal.pone.0342658

**Published:** 2026-02-13

**Authors:** Wance Wang, Jiahao Gong, Lei Yan, Bowen Xu, Yanqun Shi, Renkai Ge

**Affiliations:** 1 The School of Physical Education and Health, East China Jiaotong University, Nanchang, China; 2 School of Chemical Engineering, Shandong Institute of Petroleum and Chemical Technology, Dongying, China; 3 School of Chemistry and Chemical Engineering, Qilu University of Technology (Shandong Academy of Sciences), Jinan, China; 4 School of Biomedicine and Nursing, Shandong Institute of Petroleum and Chemical Technology, Dongying, China; Alexandria University Faculty of Medicine, EGYPT

## Abstract

Myopia has emerged as a pressing global public health issue, with a particularly sharp rise observed among school-aged children. This period represents a critical window for implementing timely, evidence-based interventions to slow myopia progression and mitigate the risk of high myopia. This study aimed to investigate the prevalence and associated risk factors of myopia among children aged 9–12 years in Shandong Province, China, with a specific focus on the roles of demographic characteristics, environmental exposures, and behavioral patterns. A cross-sectional survey was conducted in 2024 using a stratified cluster sampling design. A total of 77,629 children from 16 cities were enrolled. Data collection included standardized visual acuity assessments and structured questionnaires. Multivariate logistic regression models were used to examine the associations between behavioral factors and myopia. Only variables that showed significant associations in univariate (chi-square) analyses were included in the models, without additional adjustment for other potential confounders. The overall prevalence was 41.24%, increasing with age and reaching 44.34% at 12 years. Significant demographic risk factors included female sex (OR=1.030,95%Cl = 1.001–1.060), urban residency (OR=1.045,95%Cl = 1.014–1.077), and parental myopia, particularly when both parents were affected (OR=1.320,95%Cl = 1.270–1.372). Behavioral risk factors included reading while lying down (OR=1.093,95%Cl = 1.035–1.154), screen exposure exceeding three hours per day (OR=1.058,95%Cl = 1.010–1.109), and homework duration over three hours per day (OR=1.072,95%Cl = 1.033–1.112). Protective factors comprised outdoor activity five or more times per week (OR=0.898,95%Cl = 0.865–0.932), maintaining proper posture during reading and writing (OR=0.925,95%Cl = 0.898–0.952), screen viewing distance over three meters (OR=0.933,95%Cl = 0.905–0.961), and sleeping at least eight hours per night (OR=0.918,95%Cl = 0.876–0.961). These findings underscore the multifactorial etiology of myopia, shaped by environmental, and behavioral influences. They highlight the urgent need for comprehensive and targeted interventions, particularly for high-risk groups. This study provides robust empirical evidence to inform region-specific myopia control strategies and supports the development of public health policies aimed at improving pediatric vision health.

## Introduction

Myopia is a refractive error characterized primarily by pathological axial elongation of the eyeball, clinically manifested as reduced distance vision. It has become a major global public health concern affecting the visual health of children [1]. Epidemiological evidence indicates that children aged 9–12 years are in a critical developmental window for the onset and progression of myopia. During this period, the eye undergoes rapid growth and the refractive system exhibits high plasticity [2,3]. Concurrently, children in this age group are transitioning into secondary education, facing increasing academic demands and prolonged near work exposure [4]. Moreover, visual behavior patterns are still forming at this stage and are susceptible to both genetic predispositions and environmental influences. These characteristics render this age group an optimal target for early intervention, where timely and evidence-based myopia control strategies can effectively slow progression and reduce the risk of high myopia [5].

In recent years, the global prevalence of myopia has risen sharply. A meta-analysis by Holden and colleagues projected that by 2050, approximately 50% of the global population will be affected by myopia, with 10% experiencing high myopia [6]. The situation is particularly severe in East Asia, where estimated the regional prevalence of myopia across all age groups to reach 51.6% in 2020, 56.9% in 2030, 61.4% in 2040, and 65.3% by 2050 [6]. In China, the current prevalence of myopia among children and adolescents has reached 53.6%, with rates exceeding 80% among high school students [7,8]. Shandong Province, an economically developed coastal region in eastern China, is facing similar challenges in pediatric myopia prevention. Of particular concern is high myopia (defined as a spherical equivalent ≤ −6.00 D), which is strongly associated with an increased risk of irreversible vision-threatening conditions such as retinal detachment [9,10], glaucoma [11,12], and myopic maculopathy [13]. These complications significantly impair quality of life and can lead to permanent visual loss. Beyond its clinical implications, myopia is linked to broader social and developmental issues, including increased academic pressure, reduced outdoor activity, and negative effects on mental health, forming a detrimental feedback loop that hinders the overall well-being of children and adolescents. Moreover, the economic burden of myopia is substantial. It has been estimated that uncorrected myopia and myopic macular degeneration result in annual global productivity losses of USD 244 billion and USD 6 billion, respectively [14].

Previous studies have demonstrated that the development and progression of myopia are influenced by a combination of genetic [15,16], environmental [17,18], and behavioral factors [19]. Among these, environmental contributors such as increased near-work duration [20], reduced outdoor activity [21], and heightened academic pressure are considered key drivers behind the rising prevalence of myopia [22].

Childhood myopia has emerged as a pressing global public health challenge that necessitates multidisciplinary collaboration and systematic research approaches. This study employed a cross-sectional design to comprehensively assess the prevalence and associated risk factors of myopia among children aged 9–12 years in Shandong Province, China. The objective was to characterize the epidemiological patterns of myopia in this population and provide evidence to support the development of effective, evidence-based prevention strategies.

## Materials and methods

### Study design and participants

This study was conducted in strict accordance with the Strengthening the Reporting of Observational Studies in Epidemiology (STROBE) guidelines for cross-sectional studies [23]. To comprehensively assess the prevalence of myopia among children aged 9–12 years in Shandong Province, a large-scale cross-sectional survey was carried out from February to May 2024. A scientifically designed sampling strategy was employed to select participants from 143 primary schools across 16 cities in Shandong Province, ensuring that the sample was representative of the target population.

A two-stage sampling procedure was implemented to enhance the representativeness and reliability of the data. In the first stage, stratified sampling was conducted based on school grade to ensure balanced representation across age groups (9–12 years). In the second stage, a cluster sampling approach was applied, wherein intact classes within each grade level were defined as sampling units, and randomly selected using a probabilistic method. This multi-stage sampling framework ensured a broad demographic coverage and robust population-level representation of school-aged children in Shandong Province.

A total of 77,629 students aged 9–12 years were ultimately included in the study. Exclusion criteria comprised the presence of severe ocular diseases, evident visual impairments or deformities, and acute ocular infections or other conditions that could interfere with visual assessments. All eligible participants completed both visual acuities testing and a structured questionnaire, yielding a participation rate of 100%. The effective response rate, defined as the proportion of participants with complete and valid data, was 98.11%.

### Myopia measurement and questionnaire survey

A standardized protocol was employed for both visual acuity assessment and questionnaire administration. All investigators and participants underwent unified, systematic training to ensure consistent adherence to visual testing procedures and field epidemiological practices. Prior to data collection, the study objectives, significance, and participation procedures were thoroughly explained to students and their legal guardians.

Following the completion of the visual acuity tests, structured questionnaires were distributed to the students. To accommodate their cognitive development level, the questionnaires were completed with the assistance of parents or guardians. All completed questionnaires were collected centrally, systematically reviewed, and subjected to a double-entry procedure by two independent data clerks to ensure accuracy prior to statistical analysis.

The uncorrected visual acuity (UCVA) of students was evaluated using a standard logarithmic visual acuity chart. Refractive error was measured with an autorefractor (HRK-7000A, HUVITZ Corporation, Anshan, Republic of Korea) without cycloplegia. Myopia was defined as a UCVA of less than 5.0 (Snellen equivalent < 20/20) in either eye, combined with a spherical equivalent (SE) of ≤ −0.50 diopters (D) as measured by autorefraction under non-cycloplegic conditions. Participants meeting the diagnostic criteria for myopia in at least one eye were classified as myopic for prevalence analysis [24].

The questionnaire used in this study was developed by the authors specifically for the target population and was administered anonymously. It included questions on demographic characteristics (e.g., gender, age, family income, residential area, parental education level, and parental myopia status) as well as daily behavioral habits (e.g., frequency of using eyes while lying down or reclining, Eye use while walking or traveling (i.e., how often the child focuses on near objects, such as reading or using a phone, while walking or riding in a vehicle), frequency of outdoor physical activity, proper posture during reading and writing, distance to TV screen, distance to computer screen, daily screen time on smartphones and computers, daily TV viewing time, time spent on homework, and sleep duration). Prior to questionnaire administration, each item was explained in detail to the respondents to ensure clarity and avoid potential misunderstandings during the survey process.

Before conducting the survey, the reliability and validity of the questionnaire were evaluated. To assess reliability, the questionnaire was administered twice with a two-week interval to a subsample. The results showed no significant differences between the two administrations, indicating satisfactory temporal stability. Content validity was confirmed through expert reviews by specialists in pediatric health and epidemiology, who evaluated the relevance, clarity, and comprehensiveness of each item to ensure its appropriateness for the target population.

In accordance with the STROBE guidelines, data integrity was rigorously checked prior to analysis. Questionnaires with more than 10% missing data were excluded. For those with minimal missing data, multiple imputation was applied [23]. Independent samples t-tests (for age) and chi-square tests (for gender) were used to compare missing and complete data groups, and no significant differences were found (p > 0.05), suggesting that the missing data were randomly distributed and that the sample remained representative.

### Statistical analysis

Data were entered into a database created using EpiData 3.1 software and subjected to double data entry verification. Statistical analyses were performed using SPSS version 27.0. Continuous variables were expressed as mean ± standard deviation (±s), while categorical variables were presented as frequencies (n) and percentages (%). Descriptive statistics were used to summarize the data.

To identify potential risk factors and associations, chi-square (χ²) tests and multivariable logistic regression analyses were employed, with a significance level set at α = 0.05. Variables that showed significant differences (P < 0.05) in univariate analyses were subsequently included in the multivariable logistic regression model. The results of the multivariable logistic regression analysis were quantified using odds ratios (OR) with 95% confidence intervals (CI) to provide an estimate of the precision of the effect size [25]. To assess the goodness-of-fit and calibration of the final logistic regression model, the Hosmer-Lemeshow test was applied to ensure the robustness of the analytical framework [26].

### Ethical statement

All procedures in this study were conducted in strict accordance with relevant guidelines and regulations. Written informed consent was obtained from all participants. For minors under 16 years of age, informed consent was obtained from their parents or legal guardians. Moreover, the legal guardians of all participants provided assurance of informed consent, and signed hardcopy consent forms were collected.

This study received ethical approval from the Ethics Committee of Shandong Institute of Petroleum and Chemical Technology (registration number: KY-2024–008).

## Results

In the valid survey, the sample exhibited a balanced gender distribution, with 39,230 male participants (50.54%) and 38,399 female participants (49.46%). The age distribution was as follows: 16,007 participants were 9 years old (20.62%), 18,910 were 10 years old (24.36%), 19,803 were 11 years old (25.51%), and 22,909 were 12 years old (29.51%). The average age of the study participants was 10.6 ± 1.1 years.

### Univariate analysis of myopia prevalence across different demographic characteristics

According to the 2024 survey data on myopia prevalence in children from Shandong Province (Table 1), the overall myopia rate was 41.24%. Gender, age, residential area, family income, father’s education level, mother’s education level, and parental myopia status were found to significantly influence the myopia rate in children (P < 0.05). Specifically, the myopia rate among boys (40.85%) was slightly lower than that of girls (41.63%). The myopia rate increased progressively with age, with the highest prevalence observed among 12-year-old children (44.34%). Additionally, children with rural household registration had a significantly lower myopia rate (40.56%) compared to their urban counterparts (41.64%).

**Table 1:** Prevalence of myopia among children aged 9-12 in shandong province: results from the 2024 vision test and questionnaire survey.

Demographic characteristics	Normal Vision (%)	Myopia (%)	χ^2^	*P*
Gender			4.873	0.027
Male	23201(59.14)	16029(40.85)		
Female	22410(58.36)	15989(41.63)		
Age (year)			147.726	<0.001
9	9803(61.24)	6204(38.75)		
10	11378(60.16)	7532(39.83)		
11	11680(58.98)	8123(41.01)		
12	12750(55.65)	10159(44.34)		
Place of residence			8.625	0.003
Rural	16884(59.44)	11522(40.56)		
Urban	28727(58.36)	20496(41.64)		
Family annual income (yuan)			3.001	0.391
≤ 100,000	20957(59.04)	14542(40.96)		
100,001 ~ 200,000	15144(58.67)	10668(41.33)		
200,001 ~ 300,000	5856(58.43)	4166(41.57)		
≥ 300,000	3654(58.04)	2642(41.96)		
Father’s education level			1.967	0.374
Junior high school or below	8475(59.27)	5824(40.73)		
Senior high school	13400(58.70)	9429(41.30)		
Above senior high school	27376(58.61)	16765(41.39)		
Mother’s education level			3.393	0.183
Junior high school or below	9634(59.29)	6616(40.71)		
Senior high school	12391(58.89)	8650(41.11)		
Above senior high school	23586(58.47)	16752(41.53)		
Parents’ myopia condition			209.717	<0.001
Neither parent is myopic	21747(60.35)	14288(39.65)		
One parent is myopic	15990(59.48)	10893(40.52)		
Both parents are myopic	7874(53.52)	6837(46.48)		

The impact of family income on the myopia rate was not statistically significant (P > 0.05); similarly, the education levels of both fathers and mothers were not significantly associated with the myopia rate (P > 0.05). Children whose fathers had an education level of middle school or below exhibited the lowest myopia rate (40.73%), while those whose fathers had an education level of high school or above had the highest myopia rate (41.39%). Likewise, children whose mothers had an education level of middle school or below showed the lowest myopia rate (40.71%), while those whose mothers had an education level of high school or above had the highest myopia rate (41.53%). Parental myopia status had the most significant effect on the myopia rate (χ² = 209.717, P < 0.001). Children whose parents were both non-myopic had the lowest myopia rate (39.65%), whereas children whose parents were both myopic had the highest myopia rate (46.48%).

### Univariate analysis of factors associated with myopia prevalence

Univariate analysis results are presented in Table 2. Several behavioral factors were significantly associated with the myopia rate in children (P < 0.05). Specifically, children who frequently used their eyes while lying down or leaning forward or while walking/traveling exhibited higher myopia rates (42.85% and 44.99%, respectively) compared to those who did not engage in these behaviors (40.66% and 40.06%). Higher frequency of weekly outdoor activities and maintaining correct reading and writing posture were associated with lower myopia rates (39.70% and 40.16%), whereas children with less outdoor activity or incorrect posture showed higher rates (42.37% and 42.08%).

**Table 2:** Univariate analysis of myopia prevalence among children aged 9–12 in Shandong Province: findings from the 2024 vision test and questionnaire survey.

Independent variables	Normal vision (%)	Myopia (%)	χ^2^	*P*
Frequency of using eyes while lying down or leaning forward			14.725	0.002
Never	21973(59.34)	15055(40.66)		
Occasionally	11088(58.68)	7750(41.32)		
Often	9107(58.08)	6572(41.92)		
Always	3523(57.15)	2641(42.85)		
Frequency of using eyes while walking or riding in the car			75.750	<0.001
Never	22301(59.94)	14903(40.06)		
Occasionally	10653(58.73)	7485(41.27)		
Often	7898(57.92)	5737(42.08)		
Always	4759(55.01)	3893(44.99)		
Frequency of outdoor exercise weekly			37.300	<0.001
< 3	12267(57.63)	9019(42.37)		
3 ~ 5	18670(58.33)	13336(41.67)		
> 5	14674(60.30)	9663(39.70)		
Proper posture for reading and writing			28.867	<0.001
No	25397(57.92)	18450(42.08)		
Yes	20214(59.84)	13568(40.16)		
Distance from eyes to television screen (meter)			20.033	<0.001
< 3	15544(57.67)	11409(42.33)		
≥ 3	30067(59.33)	20609(40.67)		
Distance from eyes to computer screen (centimeter)			5.556	0.018
< 50	13837(58.13)	9967(41.87)		
≥ 50	31774(59.03)	22051(40.97)		
Screen duration on smart phone and computer daily (hour)			7.929	0.019
< 1	11528(59.59)	7817(40.41)		
1 ~ 3	27247(58.55)	19290(41.45)		
> 3	6836(58.19)	4911(41.81)		
Watching television duration daily (hour)			0.741	0.691
< 1	12526(58.90)	8739(41.10)		
1 ~ 3	17922(58.83)	12542(42.17)		
> 3	15162(58.54)	10737(41.46)		
Homework duration daily (hour)			14.070	<0.001
< 1	14137(59.69)	9547(40.31)		
1 ~ 3	17940(58.59)	12679(41.41)		
> 3	13534(58.02)	9792(41.98)		
Sleep duration daily (hour)			19.930	<0.001
< 6	5782(57.69)	4240(42.31)		
6 ~ 8	23019(58.30)	16463(41.70)		
> 8	16810(59.77)	11315(40.23)		

Additionally, greater distances to the television (≥3 m) and computer screens (≥50 cm) were linked to slightly lower myopia rates (40.67% and 40.97%) compared to shorter distances (42.33% and 41.87%). Longer daily screen time on smartphones and computers (>3 h) and longer homework duration (>3 h) were associated with increased myopia rates (41.81% and 41.98%), whereas longer sleep duration (>8 h) was linked to a lower myopia rate (40.23%). Daily television viewing time did not show a significant association with myopia (P > 0.05).

Overall, these results suggest that both visual behaviors and lifestyle factors, including outdoor activity, screen use, posture, and sleep, are associated with variations in myopia prevalence among children, highlighting potential targets for preventive strategies.

### Multivariable logistic regression analysis of myopia prevalence in children

The results of the multivariate logistic regression analysis (Table 3) indicate that, following the predefined inclusion and exclusion criteria and using a significance threshold of P = 0.05, the 13 independent variables that were significant in the univariate analysis were carefully included in the multivariate logistic regression model.

**Table 3:** Multivariable analysis of myopia prevalence among children aged 9–12 in Shandong Province: findings from the 2024 vision test and questionnaire survey.

Independent variables	*OR* (95%*CI*)	*P*
Gender		
Male	1	
Female	1.030 (1.001-1.060)	0.041
Age (year)		
9	1	
10	1.045 (1.001-1.092)	0.044
11	1.100 (1.054-1.148)	<0.001
12	1.260 (1.209-1.313)	<0.001
Place of residence		
Rural	1	
Urban	1.045 (1.014-1.077)	0.004
Parents’ myopia condition		
Neither parent is myopic	1	
One parent is myopic	1.036 (1.033-1.070)	0.032
Both parents are myopic	1.320 (1.270-1.372)	<0.001
Frequency of using eyes while lying down or leaning forward		
Never	1	
Occasionally	1.026 (0.990-1.063)	0.167
Often	1.056 (1.017-1.097)	0.005
Always	1.093 (1.035-1.154)	0.002
Frequency of using eyes while walking or riding in the car		
Never	1	
Occasionally	1.051 (1.014-1.090)	0.007
Often	1.087 (1.044-1.131)	<0.001
Always	1.224 (1.167-1.283)	<0.001
Frequency of outdoor exercise weekly		
< 3	1	
3 ~ 5	0.973 (0.939-1.008)	0.128
> 5	0.898 (0.865-0.932)	<0.001
Proper posture for reading and writing		
No	1	
Yes	0.925 (0.898-0.952)	<0.001
Distance from eyes to television screen (meter)		
< 3	1	
≥ 3	0.933 (0.905-0.961)	<0.001
Distance from eyes to computer screen (centimeter)		
< 50	1	
≥ 50	0.963 (0.934-0.994)	0.018
Screen duration on smart phone and computer daily (hour)		
< 1	1	
1 ~ 3	1.046 (1.011-1.082)	0.010
> 3	1.058 (1.010-1.109)	0.018
Homework duration daily (hour)		
< 1	1	
1 ~ 3	1.046 (1.010-1.083)	0.011
> 3	1.072 (1.033-1.112)	<0.001
Sleep duration daily (hour)		
< 6	1	
6 ~ 8	0.974 (0.931-1.018)	0.239
> 8	0.918 (0.876-0.961)	<0.001

Demographic factors such as gender, age, and residence showed significant associations: girls had a higher risk than boys (OR = 1.030, 95% CI: 1.001–1.060), risk increased progressively with age from 9 to 12 years (ORs: 10 vs. 9 years = 1.045, 95% CI: 1.001–1.092; 11 vs. 9 years = 1.100, 95% CI: 1.054–1.148; 12 vs. 9 years = 1.260, 95% CI: 1.209–1.313), and children from urban areas had higher risk compared to rural areas (OR = 1.045, 95% CI: 1.014–1.077). Parental myopia also influenced risk, with one myopic parent slightly increasing risk (OR = 1.036, 95% CI: 1.033–1.070) and both parents myopic markedly increasing risk (OR = 1.320, 95% CI: 1.270–1.372).

Behavioral factors were significantly associated with myopia risk. Frequent near-eye activities while lying down or bending over (OR = 1.093, 95% CI: 1.035–1.154) and while walking or riding in a vehicle (OR = 1.224, 95% CI: 1.167–1.283) increased risk. Conversely, frequent outdoor activities (>5 times/week) reduced risk (OR = 0.898, 95% CI: 0.865–0.932), and maintaining proper reading and writing posture was protective (OR = 0.925, 95% CI: 0.898–0.952).

Screen-related factors also affected risk. Greater distance from the television (≥3 m, OR = 0.933, 95% CI: 0.905–0.961) and computer screens (≥50 cm, OR = 0.963, 95% CI: 0.934–0.994) reduced risk. Longer daily screen time (>3 h, OR = 1.058, 95% CI: 1.010–1.109) and homework duration (>3 h, OR = 1.072, 95% CI: 1.033–1.112) increased risk. Sleep duration was inversely associated with myopia, with >8 h of sleep per day reducing risk (OR = 0.918, 95% CI: 0.876–0.961).

## Discussion

This study, based on data from the 2024 survey on myopia prevalence among children in Shandong Province, examined the associations of demographic characteristics, visual behaviors, and lifestyle factors with myopia. Among children aged 9–12 years, the overall prevalence of myopia was 41.24%. This estimate is comparable to recent reports from Eastern China, which range between 41% and 46%, but differs markedly from findings in neighboring East and Central Asian countries [27,28]. For example, in Almaty, Kazakhstan, the prevalence of myopia among schoolchildren aged 6–16 years was 28.3% (95% CI: 26.5–30.1), substantially lower than in our study population [29]. In the Ural region of Russia, the prevalence reached 46.2% (95% CI: 44.8–48.6) among children aged 6.2–18.8 years, slightly higher than the level observed in our 9–12 year age group [30]. In contrast, nationwide data from South Korea indicated a prevalence of approximately 65.4% among children and adolescents aged 5–18 years, with high myopia (spherical equivalent ≤ –6.0 D) reported in 6.9% [31], reflecting the impact of intense educational pressure and near-work activities. These comparisons highlight substantial interregional variation in the prevalence of myopia, which is unlikely to be explained by genetic background alone. Rather, environmental influences, educational systems, outdoor activity levels [32], and near-work behaviors in early childhood appear to play critical roles [33]. Moreover, these findings enhance the external validity of our study within the East Asian context, suggesting that although the prevalence of myopia among school-aged children in China is high, it is broadly consistent with regional patterns [34].

Sex was found to be significantly associated with the development of myopia, with a slightly higher prevalence observed in girls compared to boys. This finding is consistent with epidemiological evidence from multiple countries and regions. For instance, a large-scale cohort study in the United States identified female sex as an important predictor of myopia progression in children [35]. Similarly, the Generation R cohort in the Netherlands reported that girls aged 9–13 years tended to develop myopia earlier than boys [36]. In China, data from the nationwide Chinese National Survey on Students’ Constitution and Health (CNSSCH) have consistently indicated higher rates of myopia among girls across grades and regions [37]. A plausible explanation for this sex difference lies in the earlier onset of puberty among girls. Rapid pubertal growth has been shown to correlate with axial elongation and refractive changes, which may accelerate myopia progression, thereby contributing to the higher prevalence observed in female children [38,39].

Age was also identified as an important factor influencing childhood myopia. In this study, the prevalence of myopia increased significantly with advancing age, a pattern consistent with findings from several East Asian countries. A cohort study with a 4-year follow-up of primary schoolchildren in Baoshan District, Shanghai, demonstrated a substantial increase in myopia prevalence and progressive changes in refractive parameters during the transition from lower to higher grades. [40] Similarly, data from South Korea indicated that myopia is highly prevalent during the school years and enters a phase of rapid progression between 12 and 15 years of age [41]. This age-related effect may be attributable to multiple factors, including increasing academic demands, prolonged near-work activities, and the ongoing immaturity of ocular development [42]. These findings suggest that the early school years may represent a critical window for effective intervention.

Parental myopia is also an important factor influencing the development of myopia in children. In the present study, parental myopia was significantly associated with an increased risk of myopia in offspring, consistent with previous research. Several studies have reported that children with two myopic parents are at substantially higher risk of developing myopia compared to those with only one or no myopic parent [43,44]. Moreover, the interaction between genetic and environmental factors warrants attention. For example, findings from the UK ALSPAC cohort indicate that polygenic risk scores can significantly predict the risk of myopia in children, while environmental exposures, such as near-work and outdoor activities, play a critical role in modulating this risk [45]. These results suggest that parental myopia may influence the susceptibility of children to environmental exposures, thereby affecting the onset and progression of myopia.

Environmental and behavioral factors are major external drivers of the onset and progression of myopia in children. In the present study, insufficient outdoor activity and excessive use of electronic devices were both associated with an increased risk of myopia. These findings are supported by multiple studies. A randomized controlled trial in Taiwan demonstrated that an additional 40 minutes of daily outdoor activity significantly reduced the incidence of myopia among primary school children [46]. Furthermore, a systematic review and meta-analysis confirmed the protective effect of outdoor activity against myopia development [47]. Outdoor exposure is thought to promote ocular health through increased sunlight exposure, which stimulates vitamin D synthesis, while bright outdoor light induces pupillary constriction and increases depth of field, thereby slowing the onset and progression of myopia [48,49]. Conversely, prolonged near-work activities, such as reading, writing, and the use of electronic devices, have been consistently associated with myopia development [50]. This effect is thought to involve retinal signaling pathways and reduced dopamine release, which promote axial elongation, as well as the biomechanical consequences of sustained accommodation and convergence, further increasing the risk of myopia [51,52]. Furthermore, current studies have established a link between myopia and reduced time spent outdoors in children [53,54]. This behavioral pattern is hypothesized to form a negative feedback loop, wherein limited outdoor exposure contributes to myopia development, which in turn may further discourage outdoor activities [55].

Sleep has increasingly been recognized as an important lifestyle factor in myopia research. In the present study, insufficient sleep was associated with an elevated risk of myopia in children, consistent with findings from several epidemiological investigations. For instance, a study conducted in Guangzhou, China, reported that students who slept less than 8 hours per night had a significantly higher prevalence of myopia compared to those with adequate sleep [56]. Similarly, a nationwide cross-sectional study in South Korea found that shorter sleep duration was significantly associated with myopia among adolescents [57]. The underlying mechanisms may involve disruption of circadian rhythms affecting ocular development, as well as increased near-work exposure due to staying up late [58,59]. Additionally, sleep may exert a protective effect on myopia through relaxation and recovery of the ciliary muscles, thereby mitigating myopia progression. These findings suggest that ensuring sufficient sleep may play a critical role in the prevention and control of myopia in children.

Urban–rural disparities represent an important social environmental factor in the development of myopia. In the present study, the prevalence of myopia was slightly lower among rural children compared to their urban counterparts, consistent with findings from national and regional surveys [60,61]. Urban children are more susceptible to myopia due to higher academic pressures, reduced outdoor activity, and more frequent use of electronic devices [62]. In contrast, although rural children have experienced improvements in nutritional status, they generally have more opportunities for outdoor activity, which may confer some protective effect [63]. However, as lifestyle patterns in rural areas become increasingly “urbanized,” this urban–rural gap in myopia prevalence is gradually narrowing [64].

Despite providing valuable insights for the prevention and control of myopia in children, this study has several limitations. First, the reliance on self-reported questionnaires may introduce measurement bias, particularly for subjective indicators such as visual behavior. Second, non-cycloplegic refraction was used for refractive error assessment, which may lead to an overestimation of myopia prevalence, especially in younger children with stronger accommodative responses. Third, although stratified sampling was employed, the representativeness of the sample may still be affected by regional disparities. Fourth, due to the cross-sectional design, causal relationships cannot be established, and reverse causation is possible—for example, children with existing myopia may engage in different behaviors, such as increased screen time or reduced outdoor activity, which could influence the observed associations. Future studies should incorporate objective monitoring tools to obtain more accurate behavioral data, adopt longitudinal designs to track the trajectory of myopia progression, and consider broader contextual factors—such as educational environments—to strengthen the evidence base for understanding the mechanisms underlying myopia development.

The findings align with the United Nations Sustainable Development Goal 3, which emphasizes ensuring healthy lives and promoting well-being for all at all ages [65]. By providing scientific insights into improving children’s visual health and overall quality of life, this study holds both theoretical significance and practical value. Furthermore, it contributes to the global discourse on myopia control by offering data-driven evidence from China, thereby facilitating international collaboration and knowledge exchange in pediatric ophthalmic health. Clinically, our findings underscore the importance of establishing a school–community-based regular vision screening system to enable early detection and timely intervention of myopia in children. In addition, implementing public health education programs to disseminate scientific knowledge on eye care and promote healthy visual habits is essential. Personalized prevention and control strategies should be developed, with particular attention to high-risk populations. Such an integrated prevention framework not only helps curb the rising prevalence of myopia but also supports the broader goal of enhancing children’s overall health and development.

## Conclusion

This study highlights the multifactorial nature of myopia development among children, encompassing demographic characteristics, genetic predisposition, behavioral patterns, and environmental exposures. Among children aged 9–12 years in Shandong Province, the overall prevalence of myopia was 41.24%, which is comparable to recent reports from Eastern China (41–46%) and generally consistent with regional patterns in neighboring countries, though notable interregional variation exists. These findings underscore the importance of adopting a multidimensional perspective in understanding factors associated with myopia development. Factors potentially related to a lower risk of myopia, as suggested by observational evidence, include greater time spent on outdoor activities, adoption of proper visual habits, optimal viewing distance and duration of screen use, balanced academic workload, and sufficient sleep. Particular attention should be given to high-risk populations, such as girls, older age groups, urban students, and individuals with a family history of myopia. Incorporating both population-level interventions and targeted strategies for high-risk groups may provide valuable insights for mitigating the burden of myopia and promoting visual health in youth.

## Supporting information

S1 Table Questionnaire. (DOCX)

S2 Table Characteristics of questionnaire. (XLSX)

## Abbreviations

OR: odds ratio
CI: confidence interval
