# Supplementary material for: Lifestyle and environmental risk factors for myopia in children: Evidence from a large-scale cross-sectional study in Shandong, China
Source: PLoS One. 2026 Feb 13;21(2):e0342658. doi: 10.1371/journal.pone.0342658 (PMC12904384; doi:10.1371/journal.pone.0342658)
Supplement: S1 Table — (DOCX) [file pone.0342658.s001.docx]

**Supplementary Table 1** Questionnaire

questionnaire

Dear participants,

Welcome to our study, which aims to explore the current status of myopia and its influencing factors among children aged 9 ~ 12 in Shandong Province. Please take some time to answer the following questions. Please note that this is a completely anonymous questionnaire, and we will not collect any information that can identify your personal identity. There are no right or wrong answers to the questions. We hope you can respond based on your true feelings and actual circumstances.

(1) Gender

⓪ male ① female

(2) Age ___

(3) Place of residence

⓪ Rural ① Urban

(4) Family annual income (yuan)

①≤ 100000 ② 100001 **~** 200000 ③ 200001 **~** 300000 ④ ≥ 300000

(5) Father’s education level

① Junior high school or below ② Senior high school ③ Above senior high school

(6) Mother’s education level

① Junior high school or below ② Senior high school ③ Above senior high school

(7) Parents' myopia condition

① Neither parent is myopic ② One parent is myopic ③ Both parents are myopic

(8) Frequency of using eyes while lying down or leaning forward

① Never ② Occasionally ③ Often ④ Always

(9) Frequency of using eyes while walking or riding in the car

① Never ② Occasionally ③ Often ④ Always

(10) Frequency of outdoor exercise weekly

① < 3 ② 3 ~ 5 ③ > 5

(11) Proper posture for reading and writing

⓪ No ① Yes

(12) Distance from eyes to television screen (meter)

⓪ < 3 ① ≥ 3

(13) Distance from eyes to computer screen (centimeter)

⓪ < 50 ① ≥ 50

(14) Screen duration on smart phone and computer daily (hour)

① < 1 ② 1 ~ 3 ③ > 3

(15) Watching television duration daily (hour)

① < 1 ② 1 ~ 3 ③ > 3

(16) Homework duration daily (hour)

1. < 1 ② 1 ~ 3 ③ > 3

(17) Sleep duration daily (hour)

① < 6 ② 6 ~ 8 ③ > 8
